# Supplementary material for: Are carbon emissions trading and green financial instruments synergistic? -Comprehensive quantitative research based on content analysis
Source: PLoS One. 2024 Mar 7;19(3):e0298601. doi: 10.1371/journal.pone.0298601 (PMC10919601; doi:10.1371/journal.pone.0298601)
Supplement: S1 Data — (ZIP) [file pone.0298601.s001.zip › Data/地级市Z_F工作报告.zip!/║╙▒▒/║Γ╦«╩╨/└┤╫╘╣1⁄2╓┌║┼í╛┬φ┐╦╩2╛▌═°í┐_2006.docx]

各位代表：

现在，我代表枣强县人民政府向大会作工作报告，请予审议，并请县政协各位委员和列席会议的同志提出意见。

2005年工作回顾

2005年，我们是在抢抓机遇、攻坚克难、开拓创新中发展和前进的。在中共枣强县委的坚强领导下，在县人大、县政协的有力监督支持下，团结带领全县人民，牢固树立和落实科学发展观，励精图治，扎实工作，较好地完成了县十三届人大三次会议确定的各项目标任务。

我们坚持把发展作为第一要务，不断创新发展理念，完善发展思路，强化发展举措，在实际工作中注意发现解决突出问题，及时化解不稳定因素，努力营造聚精会神搞建设，一心一意谋发展的浓厚氛围，使全县经济保持了快速健康发展的好势头。现在，我向各位代表报告：2005年，全县完成生产总值51.6亿元，全社会固定资产投资29.9亿元，分别比上年增长11.5%和43%。特别是财政收入实现了历史性突破，达到3.3亿元，增长88.8%，增幅位居全市首位。一举荣膺衡水市“工业发展先进县”、“重点建设先进县”、“财政增收先进县”和“对外开放先进县”等四个荣誉称号。综合经济实力又跃上了一个新的台阶。

我们坚持实施“以工强县”战略不动摇，以园区建设为载体，以主导产业为重点，以提高竞争力为核心，强力推进工业向集群发展、向高端延伸。现在，我向各位代表报告：2005年，皮毛业新增摊点3200多家，总数达到1.8万家，新增年销售收入超千万元企业256家，总数达到374家，全年上交税金2.3亿元，增长187%；玻璃钢业新增销售收入超5000万元以上企业3家，达到12家，上交税金增长30%。以输送机械、燃气调压器为主的机械制造业方兴未艾，全年上交税金增长40%以上。玻璃钢园区建设提质提速提形象，向北扩展1.7平方公里，新上项目12个，总投资2.6亿元，入区企业累计达到68个；皮毛工业区新上项目13个，总投资1.7亿元，入区企业累计达到90 个。玻璃钢园区被国家发改委正式核准保留为省级工业园区，为长远发展创造了条件。新屯、秀屯、马屯工业小区建设开始起步，进展顺利。民营经济发展步伐加快，全年完成营业收入153亿元，上交税金3.1亿元，分别增长39%和67.7 %。工业经济强势增长，为实现更快更好发展奠定了坚实的基础。

我们坚持把项目建设作为重中之重，不断强化抓发展必须抓项目、抓项目就是抓发展的工作理念，不断加强和完善项目考核奖惩激励措施，进一步加大了对项目谋划、前期、立项、跑办、建设等环节的推进力度，特别是投入很大的精力破解土地瓶颈制约，取得了明显成效。现在，我向各位代表报告：2005年，全县谋划投资300万元以上项目120个，总投资33亿元。其中，挤进省级重点项目8个，市级重点项目22个。全年新建、续建项目85个，完成投资15.3亿元。大营国际皮草交易中心、大营污水处理厂、曼吉科工艺玻璃等一批重点项目开工建设，为经济持续发展增添了新的生机和活力。坚持把推进项目建设与改革开放结合起来，双管齐下，实施突破。全县已完成企业改制190家，占应改制企业总数的80%，共盘活资产2.45亿元，吸引民间资本1.35亿元。“招商项目年”活动扎实开展。成功举办了中国大营第十四届国际皮草交易会。全年共引进外资810万美元、内资4亿元。

我们坚持看重看大农业农村工作，注意从枣强实际出发，抓住主要矛盾和突出问题，发挥比较优势，尊重群众意愿，强力推进各项工作向深里做、向真里做、向实里做。现在，我向各位代表报告：2005年，中央制定的粮食直补、农业税减免等各项惠农政策全部落实，为农民直接增加收入1805万元。农业生产条件继续改善，完成土地整理开发1.6 万亩，新建与改造桥梁5座，新打深井102眼，建成咸淡混浇井组236个。粮食生产持续增长，全年小麦收获面积36.8万亩，总产13.9亿吨，比上年增长19%。林业、畜牧业稳步发展。全年完成造林4.28万亩、植树275 万株，新建方田林网控制面积20万亩。发展特色养殖专业户205个，年出栏超过10万只。村村通工程有保有压，共完成45.7公里，受益村36个，受益群众近5万人。文明生态村创建活动深度推进，57个村通过市验收，并涌现出东岳庄、王尧等一批先进典型。劳务输出得到加强，全县共输出2.6万人次。

我们坚持以建设环境优美城市为目标，立足当前，放眼长远，从完善规划、夯实基础、扩充功能抓起，高点站位，尽力而为，有序推进，使城镇化在高起点规划、高标准建设、高效能管理、高素质育人方面迈出了可喜的一步。现在，我向各位代表报告：2005年，城市规划工作有序展开。已聘请权威规划设计部门完成了卫千渠改造、森林公园、西、北出入口、玻璃钢园区等专项规划的编制工作。城市建设力度加大。对建设路、和平路、富强路等主干道路进行了拓宽改造，完成了10条小街小巷的路面硬化，居民出行条件得到明显改善。森林公园进入实质性建设阶段，基础设施和园林绿化成效显著。果蔬中心市场建设进展顺利，今年“五一”可正式运营。多功能住宅小区前期准备工作全部就绪，近期可开工建设。清理整治县城建设秩序初见成效。县城绿化及日常管理得到加强，绿化面积达到6.8万平方米。环卫工作向纵深开展，县城环境明显改善。经过积极争取，卫千渠排污改造工程成功立项，为今年开工建设争得了时机，奠定了基础。大营镇区改造和环境整治力度不断加大，完成了人民街、经贸路翻修改造，功能日臻完善，品位大幅提升，环境明显改观，被授予“河北省环境优美城镇”称号。交通事业谱写新篇。投资8358万元，先后完成了肃临线加宽改建、武馆线枣强南段大修工程。电力事业快速发展。城网改造工程全面完工，流常110千伏、张米、卷子、城东三座35千伏输变电站建成并投入使用。在加强硬环境建设的同时，深入开展了以“集中治理、集中服务”为主要内容的创优环境活动，收到了很好的效果。

我们坚持以人为本、富民优先，始终把增进人民福祉作为出发点和落脚点，努力做到情为民所系，权为民所用，利为民所谋，力求用工作的实际成效兑现“群众利益无小事，人民政府为人民”的庄严承诺。现在,我向各位代表报告：2005年，全县农民人均纯收入达到3691元,城镇居民人均可支配收入达到7285元,分别增长9.1%和17.7%。县政府承诺的县城中心市场建设、武馆线、肃临线建设、变电站、通信基站建设、县城路街建设、小街小巷硬化等7件实事基本完成。坚持集中财力干大事、办实事，优先考虑全局发展的需要，优先满足离退休老干部医疗费支出，优先落实公教人员政策性增资和误餐补贴，优先解决了一些如学校冬季取暖、残疾人住房、特困家庭子女升学等群众最关心、最直接、最现实的问题。

我们坚持把构建和谐枣强作为一项重大历史任务，加大了社会事业的推进力度，物质文明、政治文明、精神文明相得益彰，同频共振，经济和社会各项事业协调发展。现在，我向各位代表报告：2005年，科技工作扎实活跃。顺利通过了全国科技工作先进县复查验收，科技创新体系进一步完善，玻璃钢复合材料生产力促进中心、研发中心和皮毛研发中心三大服务平台建设积极推进，科技与经济的结合更加密切，科技对经济的贡献率达到49.6%。教育事业进步明显。学校布局调整力度加大，教育质量继续提高，高考升学率进入全市中等水平。职业教育和成人教育发展加快。顺利通过了省“普九”三轮复检。“再穷不能穷教育，再苦不能苦孩子”。我们集中三个月时间，在全县打了一场农村中小学危房改造攻坚战。投资2600多万元，新建翻建校舍2.8万平方米，维修2.9万平方米，基本消除了全县中小学危房，成为枣强教育发展史上又一空前的壮举。文化建设得到加强。坚持市场化运作，文化馆实现了整体搬迁，建成了设施完善、功能齐全的文化艺术中心。卫生事业健康发展。新型农村合作医疗工作健康运行，参合率达到75.2%，共为农民群众报免780多万元。疾控中心、县医院传染病区建成并投入运营，乡镇卫生院医疗条件得到改善。注重做好高致病性禽流感疫情防控工作。就业再就业和社会保障工作扎实推进。共争取省市再就业扶持资金160万元，全年新增就业岗位1000多个。养老、医疗、失业等保险健康运行，农村居民最低生活保障线开始启动。社区建设顺利起步，群众性精神文明创建活动进一步深化和拓展。社会稳定和安全生产工作深入开展。严格落实信访工作责任制，全面加强社会治安综合治理，有效地化解了各种不安定因素。扎实开展严打整治斗争，有力地打击了各种违法犯罪活动。高度重视并切实加强安全生产工作，全年没有发生重大安全事故。环保、计划生育、国土、统计、审计、物价等工作圆满完成任务，国防动员、广播电视、体育、档案等社会各项事业都取得新进展。

我们坚持以开展共产党员先进性教育活动为契机，高扬“树正气、讲团结、求发展”主旋律，注重加强理论学习，提高决策质量，优化资源配置，抓好工作落实，在新的实践中不断加强政府自身建设。现在，我向各位代表报告：2005年，政府职能转变步伐加快，以“提质、提速、为人民、促发展”为主要内容的机关效能建设活动全面展开，创建“学习型、创新型、服务型”机关活动不断深入。行政权力公开透明运行试点工作进展顺利。科学决策、民主决策、依法决策正在变为自觉行动。主动接受人大的法律监督、工作监督和政协的民主监督，共承办人大代表建议55件，政协委员提案75件，办结率达96 %。严格落实党风廉政建设责任制，加大了违纪违法案件查处力度。

各位代表！经过全县上下的共同努力，我们在全面建设小康、构建和谐社会的宏伟征途上迈出了坚实的步伐，实现了新的跨越。形势令人鼓舞，成绩来之不易。能够取得这样好的成绩，是县委正确领导的结果，是人大、政协强化监督、鼎力支持的结果，是全县人民团结奋斗、共同努力的结果。在此，我代表枣强县人民政府，向在各个领域和岗位上辛勤劳动、作出贡献的广大干部群众，向给予政府工作支持和监督的人大代表、政协委员和各界人士，向所有关心、支持枣强发展的朋友们，表示崇高的敬意和衷心的感谢！

在看到成绩的同时，我们也清醒地认识到，经济欠发达、实力不够强仍然是我们的基本县情，经济社会生活中的困难和问题还不少，一些长期积累的深层次矛盾尚未解决，又出现了一些不容忽视的新问题。一是在农业农村工作方面，强项不强，弱项还弱，亮点不多，农业产业化经营步履维艰，农民增收难度加大，新农村建设任务异常繁重；二是在工业方面，名品名企少、产业层次低、结构雷同度高、管理粗放失控、污染治理不到位的问题依然存在，全县南北之间、乡镇之间发展不平衡，差距逐步拉大；三是在城市建设方面，规划编制、规划控制、规划落实不够到位，管而不全、管而不严、管而不细、管而不精的问题比较突出，群众满意度还比较低；四是在构建和谐社会方面，还存在不少影响社会稳定和安全生产的隐患，涉及群众利益的不少问题还没有得到很好解决。我们还认识到，一些政府部门和工作人员缺乏大局意识、服务意识、配合意识和责任意识，思想不够艰苦，作风不够深入，工作缺乏主动性、积极性和创造性，个别工作人员弄虚作假、奢侈浪费，甚至执法犯法、贪污腐败。对此，我们一定高度重视，采取有力措施，切实加以解决。

一年来，我们在实践中深切体会到，贯彻落实科学发展观，推进枣强更快更好发展，必须把握发展这条主线，必须贯穿创新这个灵魂，必须践行为民这个宗旨，必须遵循法纪这个准绳。只要我们一以贯之地坚持下去，就一定能够乘势而上，不断开创政府工作的新局面。

2006年主要任务

对于枣强来说，2006年是一个特殊的年份，改革发展的任务重于以往任何一年，需要办的事情多于以往任何一年，亟待研究解决的矛盾和问题难于以往任何一年，领导和群众对我们工作的期望值高于以往任何一年。这些都告诉我们，做好今年的工作，意义非同寻常，必须进一步增强忧患意识、创新意识和发展意识，必须充分做好应对各种困难和挑战的思想准备，必须在开拓创新、真抓实干上付出加倍再加倍的努力。年初召开的县委八届四次全会高瞻远瞩，审时度势，提出了“围绕一个总目标，牢牢把握六项原则，大力实施六项工程，努力构建和谐社会，实现经济社会全面协调可持续发展”的总体目标、思路和要求。今年的政府工作，就是要紧紧围绕这一总体目标、思路和要求，创造性地抓好落实，务求取得新的成效、新的突破。

主要预期目标是：地区生产总值增长13 %以上，全部财政收入增长15 %以上，全社会固定资产投资增长25 %以上，城镇居民可支配收入增长10 %以上，农民人均纯收入增长 6%以上。

今年，要着力抓好以下六方面的工作：

一、精心组织实施好主导产业提升工程。皮毛、玻璃钢两大主导产业是全县经济的重要命脉，是富民强县的重要保障，促其更快更好地发展，是历史的选择，是人民的选择。无论在前进的道路上遇到什么艰难险阻，都必须坚定不移地向前推进。

（一）要用力提升主导产业的竞争力。这是立于不败之地，实现可持续发展的根本所在。为此，要突出抓好“四重”：一是重视引导。重视加强对主导产业发展的引导和扶持是我们的一贯思想和做法。这些年，县委、县政府先后出台了一系列有效管用的政策措施，凡是没有明文废止的，都要不折不扣地贯彻执行。根据发展的需要，今年我们围绕鼓励支持优势企业做大做强，又研究制定了十二条政策性规定。其目的就是通过发挥政策的导向作用，把广大业主的注意力和积极性集中到把企业做大做强上来。二是重点突破。提升主导产业竞争力是一项系统工程，必须整合力量，实施重点突破。要以打造“中国裘都”、“中国毛皮产业基地”为目标，择优筛选100家骨干企业，组织引领这些企业在膨胀企业规模、提高装备水平、推动技术创新、实施争创名牌名企名家战略、开拓国内外市场等方面进行重点突破，力争年内产销过亿元企业达到12家，新增高新技术企业4家、高新技术产品5个，培育市级以上名牌5个，3家企业佩带皮毛真皮标志。三是重心下移。提升主导产业竞争力，固然离不开企业自身的努力，同时需要全社会的关注和支持。县委、县政府将2006年确定为“创业服务年”。各级各部门都要将工作触角和服务重心下移，为企业做大做强尽职责、搞服务、创环境、做贡献，努力在全县形成做大做强企业的强大攻势。四是重拳出击。从总体上看，今年皮毛、玻璃钢两大主导产业发展面临的有利条件很多，但制约发展的矛盾也不少，还有一些不确定因素，需要采取正确的应对措施，防止出现大的起落。我们强调创优发展环境，并不是放纵胡作非为。现在，一些个别企业经营者利益熏心、唯利是图、弄虚作假、违法行事，严重扰乱了正常的经济秩序，给产业的发展和枣强形象造成了极坏的影响。对此，务必引起高度重视，打好组合拳。要加大法制、政策的宣传教育力度，加大环境创优力度，加大污染治理力度，尽最大的努力规范产业的发展。同时，对各种违法行为，要敢于重拳出击，依法严厉打击，决不姑息迁就。

（二）要用力提升项目建设的支撑力。项目是发展之源、增长之基。加强项目建设仍然是政府工作的重中之重和当务之急。继续贯彻“全党抓经济、重点抓工业、关键抓项目、突出抓园区”的战略方针，认真总结汲取以往的经验教训，坚持“四措并举”，做到“三个集中”。坚持四措并举，就是坚持项目谋划与做大做强主导产业并举、做好项目前期与争列省市重点并举、争列省市重点与保障土地供应并举、加大投入与招商引资并举。这四个并举相互制约，必须环环相扣，抓紧抓好。做到三个集中，就是加速推进工业向园区集中、园区向县城集中、农民向城镇集中。具体讲， 一是在建项目要抓紧进度。对银河裘服、中韩合成新材、生皮市场、卫千渠改排等107个在建项目，严格落实县领导、主管部门、金融单位、项目法人“四位一体”责任制，加强协调调度，倒排工期，挂图作战，确保建设进度和工程质量。二是项目谋划要力求深度。聘请权威专家和业内人士，依托主导产业和骨干企业谋划一批重大项目和战略支撑项目，下功夫把各项前期工作做深做细，为项目核准和建设创造条件。力争全年谋划、建设重点项目135个，总投资31.5亿元。三是项目跑办要再增力度。严格落实领导分包项目责任制，卡死责任，盯死跑紧，千方百计挤进省市计划笼子。全年争列省级重点项目12个、市级重点项目20个。特别要千方百计突破土地瓶颈制约，确保项目建设特别是重点项目建设用地需要。

（三）要用力提升工业园区的承载力。玻璃钢、皮毛两个工业园区已经成为我县经济发展的平台、城市建设的亮点、对外开放的窗口。但从发展的角度看，其承载能力远不能满足需要。作为一项紧迫而且事关全局的重大任务，今年要着力抓好三件事：一是抓好提升。年内玻璃钢园区要完成胜利北路、工业街、富强北路、朝晖街硬化、绿化和排水工程；皮毛工业区要完成一纵二横路街建设，完善功能，提升品位。严把项目入区关，提高项目科技含量、投入强度和集约开发水平。玻璃钢园区新建续建项目30个，完成投资3.8亿元；皮毛工业区新建续建项目35个，完成投资1.9亿元。二是抓好扩容。要抓紧对下一步两个园区发展进行规划论证，超前做好土地调规和修编工作，择机启动建设，为项目进区提供新的空间。三是抓好增效。建设园区，效益第一。要抓紧研究根治税收流失的政策措施，提高园区特别是玻璃钢园区对财政的贡献率。与此同时，要加大新屯、秀屯、马屯、枣强镇四个工业小区的推进力度，按照“统一规划、分步实施、各有特色、错位发展”的原则，强力招商引资，确保尽快取得成效。全力打造以县城、大营两大园区为龙头、产业结构合理搭配、准入门槛高低有别、投资主体互为补充的园区发展新格局。

（四）要用力提升对外开放和招商引资的拉动力。“开放兴县”是县委、县政府确定的两大主体战略之一。要以深入实施“招商项目年”活动为载体，进一步增强开放意识、提高开放本领、创造开放环境、扩充开放实效。一要靠全民招商引资。进一步完善和落实招商引资的政策措施，大力推进产业招商、特色招商、骨干招商、品牌招商、环境招商、以商招商，组织好针对日韩、欧美、珠三角、长三角、港澳台等国家和地区的招商推介活动。办好首届中国裘皮制品及原料、半成品采购定货会和第十五届中国大营国际皮草交易会，不断推进招商引资工作向高层次、宽领域发展。二要靠服务亲商投资。注意加强与客商沟通，倾听客商意见和呼声，改进服务措施，兑现政策承诺，塑造“可信、可交、可靠”的诚信形象，不断坚定客商投资的信心和决心。三要靠环境安商稳资。以保护客商权益为重点，严厉打击扰商、欺商、排商等行为，设身处地为客商排忧解难，真正使投资者安心、舒心、放心。四要靠回报富商扩资。牢固树立富己先富商、富商必富县的理念，在政策范围内最大限度地让利于商，激发客商连续投资、追加投资的积极性。力争全年引进内资4.6亿元，实际利用外资980万美元。同时，切实抓好外贸出口工作，进一步调整出口方略，优化出口结构，规范出口行为，促进外贸出口稳定增长。

二、精心组织实施好社会主义新农村建设工程。这是一项新的重大历史任务，序幕已经拉开。我们必须紧跟时代前进的步伐，从解决薄弱环节和突出问题入手，有的放失地展开工作，尽快使广大农村面貌有比较明显的变化。一是在用足用好惠农政策上要有新作为。可以断定，今年和今后一段时间，将是国家惠农政策集中出台而且力度不断加大的一个新的机遇期。谁能用足用好这些政策，谁就会在新一轮发展中争取主动，实现跨越。因此，我们必须增强敏锐性，提高执行力，放大政策功效。一方面，要按照中央和省市要求，将各项惠农政策不折不扣地落实到位，确保不滞留、不走样；另一方面，要及时掌握第一手信息，加强对策研究，加大争取支持的跑办力度，力争更多政策能够惠及枣强百姓。二是在发展农村生产力上要有新成效。落实建设新农村五句话20字的要求，经济是基础。夯实这个基础，必须切实加强农田水利建设，不断改善农业生产条件；必须加快发展现代农业，促进小麦、棉花、林果、畜牧四业稳定发展和农民持续增收；必须加强农业技术推广和服务，不断提高农业创新和转化能力；必须强力推进农业产业化经营和劳务输出，多渠道增加农民收入。今年，全县打更新井100眼，建咸淡混浇井组200个，粮食总产稳定在5.5亿斤以上，完成农业开发20万亩，植树200万株，新增方田林网控制面积18万亩，果品年产量达到6万吨，特色养殖存栏达到42万只，实现劳务输出2.7万人次。三是在农村公路建设上要有新突破。要想富，先修路。建设新农村同样是这个道理。“寸步难行”什么事情也干不成干不好。基于此，今年我们将加大农村公路建设的投入力度。首先，加大对东北部经济欠发达地区的扶持倾斜力度，全面完成枣景路建设，力争完成肖（张）马（朗）路建设。同时，加大对东南部经济集中地区的扶持倾斜力度，全面完成皮草环线、张唐路建设。这4条主干线建设总里程122公里，途经10个乡镇100多个村，惠及近十万人民群众。完成62个村52公里“村村通”工程建设，构建起主干相通、支线相连、纵横交织、便捷顺畅的大交通网络，畅通城乡间的人流、物流、信息流，为农民增收致富铺就康庄大道。四是在文明生态村建设上要有新进展。创建文明生态村是建设社会主义新农村的有效抓手。今年要按照“科学规划、突出重点、联动共建、连片发展”的思路，巩固提高第一批，拓展推开第二批。从今年开始，要组织专业人员，着手编制村镇建设和发展规划，使农村的各项建设在规划的指导下有序推进。进一步丰富创建内涵，把创建活动与发展生产、改变村容村貌、提高村民素质和生活水平有机结合起来。突出抓好文明生态示范村的卫生环境管理、村规民约建设和文明农民教育工程。同时，要加强农村公共服务体系建设，切实解决农民看病难、看病贵等实际问题。

三、精心组织实施好省级环境优美城市创建工程。今年是创建省级环境优美城市的第一年。根据创建需要和群众要求，在认真调查研究的基础上，确定启动实施9项城建工程。主要包括：①全长2.2公里的卫千渠县城段改造升级和总长1.5万米的县城污水改排工程；②多功能住宅小区一期建设工程；③西出口、北出口、南出口拓宽改造工程；④胜利路、人民街、裕华街便道硬化工程；⑤建设路、胜利路、新华街绿化工程；⑥原煤建北街等5条小街小巷及建设路南段路面硬化工程；⑦县城第二供水厂建设工程；⑧森林公园二期建设工程；⑨枣强中学新校区即平原中学建设工程。这九项重点工程完成后，对完善城市功能、提升城市品位、优化城市环境、塑造城市形象，必将产生积极而重要的推动作用。考虑到今年县城建设开工项目比较多、建设战线比较长、投资强度比较大、工作任务比较重，我们将在进一步强化组织领导、明确责任分工的前提下，特别要严格把好规划设计、工程招标、施工质量和时间进度四道关口，力求把每项工程建设成为百姓满意的“民心”工程、社会认可的精品工程和经得起考验的阳光工程。要把城市管理工作放在更加突出的位置，标本兼治，整体推进。一要强化宣传教育，提高全民素质，加快实现城市群体由农民向市民的转变；二要学习借鉴成功经验，研究制定管理办法，加快实现由以人治为主向以法治为主的转变；三要坚持高标准、严要求，全覆盖，加快实现由粗放型管理向精细型管理的转变；四要强化职能，整合力量，组织开展对主干路街广告牌匾、沿街墙体立面和拆违拆破、清理马路市场等专项治理活动，加快实现由临时突击向建立长效机制的转变，努力将城市管理提高到一个新水平。大营镇是全县的经济重镇，要按照“抓规划、扩功能、提品位、强管理”的思路，进一步加大工作力度，加速向现代化小城市迈进。

四、精心组织实施好体制改革创新工程。通过改革创新，使各项工作充满生机和活力，是今年政府工作的“重头戏”。一要积极稳妥地推进企业改制。对慧星调压器总厂、针织厂等11家已经进入改制程序的企业，抓紧进行资产处置，妥善安置职工，确保上半年完成改制任务。对商业、物资、县社系统51家尚未进入改制程序的企业，要坚持因企制宜，分类指导，依法采取破产、重组、股份制改造等多种形式，科学制定改制方案，充分征求职工意见，严格规范操作程序，确保改制工作顺利进行。对尚未改制到位的乡镇集体企业，要加大督导力度，加快改制步伐，限期完成任务。对已经完成改制任务的企业来一次回头看，引导企业进一步完善法人治理结构，向现代企业迈进。二要继续深化农村改革。要坚持长期稳定和不断完善农村基本经营制度，切实保护农户的土地承包经营权，保障农民的物质利益和民主权利。进一步健全农产品市场体系和城乡统一的要素市场体系。按照上级要求，积极推进农村金融体制改革、土地征用制度改革和粮食流通体制改革。通过改革，不断增强农村发展的动力和活力。三要加快行政管理体制改革步伐。按照上级统一部署，本着积极稳妥、循序渐进的原则，以政企分开、政资分开、政事分开、政府与市场中介组织分开为目标，加快政府职能转变，深化行政审批制度改革，构建政府公共服务框架。加快教育、卫生等事业单位人事制度改革步伐，逐步建立以聘用制为中心，人员能进能出、职务能上能下、待遇能高能低的新型人事制度。要以“行为规范、运转协调、公正透明、廉洁高效”为目标，探索建立乡镇行政管理体制和运行机制的有效途径，促进乡镇职能转轨变型。

五、精心组织实施好创优环境工程。发展选择环境，环境决定发展。一要努力营造一流的领导环境。加快发展是全县人民的共同利益，是全县上下的共同责任。无论是领导干部还是一般干部，无论是经济部门还是非经济部门，无论是县直部门还是基层乡村，都要在经济发展中承担责任、落实义务，都要为加快发展尽心出力、献计献策，都要自觉做到部门利益服从整体利益、局部利益服从大局利益，切实做到一切从发展着想，一切为发展让路。二要努力营造一流的服务环境。政府各部门及工作人员都要把服务群众作为第一责任，把服务企业做为第一对象，真正做到支持不刁难、帮忙不索取，对基层、对企业、对群众在发展中遇到的问题，该办的要坚决办，不能等、不能拖、更不能推，努力为经济发展提供全方位优质服务。三要努力营造一流的政策环境。进一步落实完善加快发展的各项政策措施，保持政策的连续性和实用性。要加大政策的宣传力度，增强透明度，确保政策落实到位。四要努力营造一流的创业环境。旗帜鲜明地支持创业者、爱护改革者、批评空谈者、惩治诬陷者、宽容失败者，大力倡导创业、创优、创新精神，努力营造干事创业的良好氛围。五要努力营造一流的信用环境。依法强化市场监管，严厉打击各种坑蒙拐骗、欺行霸市、敲诈勒索等不法行为，建立守信激励和失信惩戒机制，搭建公正、公平、平等竞争的市场平台。

六、精心组织实施好社会进步和社会和谐工程。以造福人民为根本 ，大力发展社会事业，让广大人民群众共享发展成果。一要积极推进科技进步。要不断巩固和发展科技工作先进县成果，加强与大专院校、科研单位的密切合作，坚持面向经济主战场，切实增强科技创新能力和主导产业竞争能力。二要优先发展教育事业。统筹城乡教育发展，优化学校布局，深化教育改革，改善办学条件，努力提高教育质量和办学水平；大力兴办职业教育，依托全县优势产业，推进校企联合，培养实用人才，为经济和社会发展提供人才支撑。认真落实国家对农村教育的各项政策，加大对困难家庭子女帮扶力度，确保不让一名学生因贫辍学。三要切实加强卫生工作。加大新型农村合作医疗工作力度，落实补助标准，努力实现农民参合率和报免率“双提高”。加强医院、卫生院和社区服务站建设，改善医疗条件，提高医疗水平。建立突发公共卫生事件应急机制，不断提高疾病预防和控制能力。四要努力做好就业再就业和社会保障工作。加强就业再就业服务体系建设，广辟就业岗位，确保将登记失业率控制在4%以内。加强社会保险体系建设，使养老、医疗、失业等各项社会保险基金总额和参保人员稳步增长。加强社会救助体系建设，健全并落实城乡最低生活保障制度，切实解决好弱势群体的生产生活问题。五要全力维护社会稳定。加强信访工作，深化社会治安综合治理，深入开展打黑除恶专项行动，依法严厉打击各类刑事犯罪活动，确保社会稳定。要严格落实安全生产责任制，深入开展安全生产专项整治，坚决遏制重特大事故的发生，确保人民群众生命财产安全。六要扎实推进精神文明建设。以大力倡导“八荣八耻”的社会主义荣辱观为重点，以深入开展“五创”活动为载体，加强职业道德、社会公德和家庭美德教育，不断提高公民素质和社会文明程度。此外，认真做好国防动员、计划生育、统计、审计、物价、档案、邮政等工作。

关于《国民经济和社会发展

第十一个五年规划纲要(草案)》的说明

今天提请大会审议的《国民经济和社会发展第十一个五年规划纲要(草案)》，明确了我县未来五年国民经济和社会发展的奋斗目标、指导方针和主要任务，是指导经济社会发展的纲领性文件。下面，我就几个问题作简要说明。

一、“十一五”规划《纲要(草案)》的编制过程。县委、县政府对制定“十一五”规划工作高度重视。从去年5月份开始，就成立了纲要起草工作领导小组，县四套班子领导同志及相关部门做了大量深入细致的调查研究，为编制《纲要(草案)》提出了许多指导性意见。之后，县委、县政府多次召开专题会议，对《纲要(草案)》进行认真的研究和讨论，并广泛征求了各乡镇、部门和社会各界人士的意见。经过反复修改，形成了《纲要(草案)》。可以说，《纲要(草案)》形成的过程，是充分发扬民主、发挥集体智慧的过程，也是统一思想、形成共识的过程。

二、“十五”期间国民经济和社会发展主要情况。《纲要(草案)》指出，过去的五年，全县人民坚持以邓小平理论、“三个代表”重要思想和科学发展观为指导，扭住发展第一要务不放松，努力克服前进道路上的各种困难，与时俱进，开拓创新，顺利完成了“十五”各项目标任务，经济和社会发展取得了令人瞩目的成就。主要表现在：一是综合实力上了一个大台阶，一跃挤入全市先进行列；二是结构调整迈出新的步伐，以工强县取得重要进展；三是发展后劲明显增强，城乡面貌大为改观；四是改革开放步伐加快，经济发展活力迸发；五是社会事业全面进步，人民生活水平稳步提高。其它各项事业、各项工作也都取得可喜的进步和成绩。总之，这五年是不平凡的五年，是枣强发展实现重要转机、开始起步前进的五年。五年来，我们不仅收获了物质财富，而且积累了宝贵的工作经验。对此，《纲要(草案)》都作了分析和说明。

三、“十一五”时期经济社会发展的指导原则和主要目标。《纲要(草案)》强调，未来的五年，是枣强全面建设小康社会和率先发展的关键阶段。站在新的历史起点上，我们既面临着新的机遇，又面临着新的挑战。只要我们紧紧抓住机遇，主动迎接挑战，未来五年的发展大有可为。总的指导思想是：高举邓小平理论和“三个代表”重要思想伟大旗帜，坚持以人为本，坚持以科学发展观统领全局，坚持把发展作为第一要务不动摇，立足于“快”（抢抓机遇，加快发展），着眼于“好”（实现经济社会的全面、协调、可持续发展），主攻于“高”（提高经济运行质量、转变经济增长方式），落实于“强”（晋位争强），实施“两大战略”（工业强县、开放兴县），推进“三化”进程（新型工业化、城市化和农业产业化），突出“四项建设”（项目建设、园区建设、城镇建设、社会主义新农村建设），促进物质文明、政治文明、精神文明的全面进步，建设更加富强、更加和谐、更加美好的新枣强。

据此，《纲要(草案)》提出了我县“十一五”时期经济社会发展的主要目标：全县生产总值年均递增13 %以上，固定资产投资年均递增25%以上，财政收入年均递增15%以上，在全市率先发展，综合实力跻身全省前三十强。同时也明确了人民生活、城乡建设、社会发展、资源效率等方面的目标。这些目标符合枣强实际，经过努力是能够实现的。下面，我重点就经济发展速度说明如下：全县生产总值年均增长13%以上，符合我县近年来的发展趋势。“十五”时期，全县生产总值年均增长10.3%，并呈现平稳加快的态势，尤其是近年来，通过大力实施项目带动战略，增上了一大批支撑能力强的项目，这些项目在“十一五”期间将发挥更大的效益，成为现实的经济增长点。因此，按照目前的发展趋势和未来五年各方面的发展条件，“十一五”时期保持13%的增速，是可以实现的。《纲要(草案)》没有把目标定得更高，主要基于以下考虑：一是有利于把主要精力放在转变经济增长方式、提高经济增长质量和效益上来；二是我县主导产业外向度高，易受国际不确定因素影响，应留有余地；三是由于我县生产总值基数较大，要实现到2010年人均生产总值比2000年翻一番的目标，包括人口增长因素，“十一五”时期生产总值年均增长只要达到7.8%即可实现。因此，确定13%的增长目标既积极又稳妥，体现了科学发展的要求。

四、“十一五”时期的战略重点和主要任务。《纲要(草案)》明确指出了“十一五”时期的五项主要任务：一是以工业经济为主导，以项目建设为支撑，倾力打造“实力枣强”；二是以农业增效、农民增收为目的，以建设社会主义新农村为主线，倾力打造“富庶枣强”；三是以招商引资为重点，以强化措施为保证，倾力打造“开放枣强”；四是以“硬环境”建设为基础，以“软环境”建设为保障，倾力打造“魅力枣强”；五是以统筹发展为核心，以“三个文明建设”为目标，倾力打造“和谐枣强”。这“五个枣强”，既是工作重点，也是努力方向。实现“五个枣强”，是枣强发展的战略选择，是形势发展的必然要求，也是全县人民的共同愿望。对如何推进好“五个枣强”建设，《纲要(草案)》进行了明确具体的阐述。需要说明的是，这五个方面密切关联、相辅相承，在实际工作中必须把握全局，突出重点，统筹推进。

各位代表！“十一五”将是枣强发展史上的黄金时期，宏伟的蓝图已经展现在我们面前。我们坚信，勤劳智慧的枣强人民，一定会把这个宏伟的蓝图变为现实，我们的事业必将更加兴旺发达，我们的家园必将更加美丽富饶，我们的生活必将更加幸福安康。

今年是“十一五”规划的开局之年，也是我们向全省三十强迈进的起步之年。新的形势和任务，对政府工作提出了更高的要求。我们必须全面加强自身建设，奋发有为地做好新一年的政府工作。

做好新一年的政府工作，必须坚持以科学发展观为指导，努力提高决策质量和水平。实践证明，决策正确，事半功倍；决策失误或错误，就会因难于落实而贻误时机，甚至给党和人民的事业造成重大损失。在这个时候，我们既耽误不起，更失误不起。因此，要更加重视科学决策、民主决策和依法决策，特别是重点工作、重要事项和重大投资，都必须经过集体研究决定。同时，要下大力抓好决策落实。对确定的事情和工作，都要定人员、定责任、定时间、定进度。通过建立和落实工作责任制，切实解决“责任不清、压力不足”的问题，坚决克服那种看似都在抓实则没人抓、看似都在管实则没人管、看似都在负责实则没人负责的不良现象。

做好新一年的政府工作，必须始终保持昂扬向上的精神状态，把全部心智放在“干事、创业、为民”上来。我们知道，在一年的时间内，不可能所有工作都在全市、全省乃至全国领先，但完全可以而且应该通过努力，后来居上，在某些方面、某些工作上跻身前列。我们还知道，在工作中没有轻松和现成的路可走，还会遇到这样那样的困难和问题。但我们决不会因成绩而停步，遇困难而退缩，被矛盾所困惑。我们希望所有政府工作人员都要抓住每一天时间，动足每一根脑筋，激活每一个细胞，凝聚每一份智慧，把握每一个机会，调动每一份力量，办好每一件实事，争取每一次成功。

做好新一年的政府工作，必须真抓实干，并力求使之成为一种工作常态和自觉行动。为政之道贵在真抓实干。要大力弘扬崇尚实干、不事张扬的传统和作风，真正把劲用在推动工作落实上，用在发现问题、研究问题和解决问题上，用在提高工作标准和效能上，用在打基础、增后劲、办好关系百姓利益的事情上，坚决不搞不得人心的形象工程和劳民伤财的政绩工程。

做好新一年的政府工作，必须牢记执政为民的宗旨，尽心竭力为群众办实事、解难事、做好事。今年，要力争办好以下9件实事：①完成卫千渠县城段改造升级工程；②启动县城多功能住宅小区一期工程建设；③力争完成枣景路一期工程、张唐路、皮草环线建设，启动肖（张）马（郎）路建设；④完成森林公园二期工程建设；⑤完成原煤建北街、仿古街、老礼堂街等5条小街小巷及建设路南段路面硬化工程；⑥启动胜利路、人民街、裕华街便道硬化工程；⑦启动西出口、北出口、南出口拓宽改造工程；⑧完成大营皮毛工业区、臣赞、石村三个35千伏输变电站建设，新建45个通讯基站；⑨启动枣强中学新校区即平原中学建设工程。

做好新一年的政府工作，必须坚持从严治政，深入开展廉政建设和反腐败斗争。要认真实施《公务员法》，加强公务员队伍教育、管理和监督，提高公务员队伍素质。政府工作人员特别是领导干部都要忠于职守，勤勉尽责，全心全意为人民服务；都要顾全大局，加强纪律性，做到令行禁止，不折不扣地执行国家的法律法规、方针政策；都要求真务实，力戒空谈，克服官僚主义、形式主义、弄虚作假和浮夸作风，把各项任务和部署真正落到实处。要全面落实党风廉政建设责任制，建立健全教育、制度、监督并重的惩治和预防腐败体系。加强行政监察和审计监督，严厉查处违法违纪案件。需要强调的是，枣强县是全市第一个行政权力公开透明运行试点县。在去年工作的基础上，今年要在政府各部门深入展开。要充分运用各种载体公布职权目录和流程图，实现行政权力运行的动态公开，使行政权力运行的各个环节置于阳光操作之下，决不给乱作为者提供机会，也不给不作为者留下空隙，坚决杜绝商业贿赂和腐败现象的滋生。要扩大群众的知情权、参与权和监督权，更加主动自觉地接受人大的法律监督、政协的民主监督和社会各界监督。

各位代表！光荣而又艰巨的任务已经历史地落在我们肩上。让我们更加紧密地团结起来，在中共枣强县委的领导下，高举邓小平理论和“三个代表”重要思想伟大旗帜，全面贯彻落实科学发展观，万众一心，开拓创新，为顺利实现既定目标任务，书写枣强率先发展、和谐发展、更快更好发展的新篇章而努力奋斗！
